# Development of a scalable mental healthcare plan for a rural district in Ethiopia

**DOI:** 10.1192/bjp.bp.114.153676

**Published:** 2016-01

**Authors:** Abebaw Fekadu, Charlotte Hanlon, Girmay Medhin, Atalay Alem, Medhin Selamu, Tedla W. Giorgis, Teshome Shibre, Solomon Teferra, Teketel Tegegn, Erica Breuer, Vikram Patel, Mark Tomlinson, Graham Thornicroft, Martin Prince, Crick Lund

**Affiliations:** **Abebaw Fekadu**, MD, PhD, MRCPsych, Department of Psychiatry, Addis Ababa University, Addis Ababa, Ethiopia, Department of Psychological Medicine, Centre for Affective Disorders, Institute of Psychiatry, Psychology and Neuroscience and Centre for Global Mental Health, Health Services and Population Research Department, King's College London, London, UK; **Charlotte Hanlon**, PhD, MRCPsych, Department of Psychiatry, Addis Ababa University, Addis Ababa, Ethiopia and Centre for Global Mental Health, Health Services and Population Research Department, King's College London, London, UK; **Girmay Medhin**, PhD, Institute of Pathobiology, Addis Ababa University, Addis Ababa, Ethiopia; **Atalay Alem**, MD, PhD, **Medhin Selamu**, MA, Department of Psychiatry, Addis Ababa University, Addis Ababa, Ethiopia; **Tedla W. Giorgis**, PhD, Federal Ministry of Health of Ethiopia, Addis Ababa, Ethiopia; **Teshome Shibre**, MD, PhD, **Solomon Teferra**, MD, PhD, **Teketel Tegegn**, MD, Department of Psychiatry, Addis Ababa University, Addis Ababa, Ethiopia; **Erica Breuer**, MPH, Alan J Flisher Centre for Public Mental Health, Department of Psychiatry and Mental Health, University of Cape Town, Cape Town, South Africa; **Vikram Patel**, PhD, MRCPsych, Centre for Global Mental Health, London School of Hygiene and Tropical Medicine, London, UK, Centre for Mental Health, the Public Health Foundation of India and Sangath, Alto-Porvorim, Goa India; **Mark Tomlinson**, PhD, Stellenbosch University and University of Cape Town, Cape Town, South Africa; **Graham Thornicroft**, PhD, FRCPsych, **Martin Prince**, MD, FRCPsych, Centre for Global Mental Health, Health Services and Population Research Department, King's College London, London, UK; **Crick Lund**, PhD, Alan J Flisher Centre for Public Mental Health, Department of Psychiatry and Mental Health, University of Cape Town, Cape Town, South Africa, and Centre for Global Mental Health, Institute of Psychiatry, Psychology and Neuroscience, King's College London, UK

## Abstract

**Background**

Developing evidence for the implementation and scaling up of mental healthcare in low- and middle-income countries (LMIC) like Ethiopia is an urgent priority.

**Aims**

To outline a mental healthcare plan (MHCP), as a scalable template for the implementation of mental healthcare in rural Ethiopia.

**Method**

A mixed methods approach was used to develop the MHCP for the three levels of the district health system (community, health facility and healthcare organisation).

**Results**

The community packages were community case detection, community reintegration and community inclusion. The facility packages included capacity building, decision support and staff well-being. Organisational packages were programme management, supervision and sustainability.

**Conclusions**

The MHCP focused on improving demand and access at the community level, inclusive care at the facility level and sustainability at the organisation level. The MHCP represented an essential framework for the provision of integrated care and may be a useful template for similar LMIC.

Despite ongoing methodological challenges, epidemiological estimates are now available on the magnitude of unmet needs among the mentally ill in low- and middle-income countries (LMIC).^1–5^ The main gap in knowledge is on how best to meet these needs. Prompted by his first-hand experience of working in Ethiopia, the late Professor Robert Giel made the argument over four decades ago, well before the Alma Ata declaration, that if mental health services are to be expanded to address the mental health needs in LMIC, two things have to happen: (a) certain disorders have to be prioritised as targets of intervention given the resource constraints in LMIC; and (b) mental healthcare has to be provided by non-specialists through task-sharing and integration into primary care.^6^ In recognition of the ongoing pertinence of these recommendations, the Ethiopian Ministry of Health is committing itself to the integration of mental healthcare into primary care. Three key recent initiatives support the accelerated integration of mental healthcare: (a) the latest Health Sector Development Plan (HSDP-IV) published in 2010 proposes increasing the proportion of health facilities providing integrated mental healthcare to 50%;^7^ (b) the Federal Ministry of Health has piloted the World Health Organization (WHO) Mental Health Gap Action Programme (mhGAP) in selected sites in four regions of the country;^8^ and (c) the main road map for the scale up of mental health services in the country has been the launch of the national mental health strategy by the Federal Ministry of Health just over a year ago.^9^ The strategy mandates explicitly the integration of mental healthcare into every primary care facility in the country.

However, evidence on the best approaches to support the practical integration of mental healthcare into primary care is still required. Key questions include the following. What kind of organisational (system-level) support is needed and what interventions are required to gain this organisational support? What kind of capacity strengthening support is required to enable primary care staff to provide safe, effective and inclusive care? What are the best approaches to improve accessibility of care in a traditional rural population that live in difficult to access terrains? This paper aims to answer these questions by describing a mental healthcare plan (MHCP), which defines the key intervention packages to be implemented in one rural district in Ethiopia.

## Method

The study was conducted as part of the PRogramme for Improving Mental health carE (PRIME), which is a research consortium involving five LMIC: Ethiopia, India, Nepal, South Africa and Uganda. The aim of PRIME is to develop evidence on the best approaches for integrating mental healthcare into primary care in LMIC.^10^

### The Ethiopia study setting

The setting for the implementation of the MHCP is the Sodo district, Gurage Zone, Southern Nations, Nationalities and Peoples Region (SNNPR) (online Fig. DS1), located about 100 km south of the capital city, Addis Ababa. The total population is 161 952 people (79 356 men and 82 596 women) with about 90% living rurally.^11^ The district is geographically diverse with some of the most inaccessible terrains in the region. It has the second highest population and the largest surface area of any district in the SNNPR. Amharic is the official language of the district, as is the case for the region. The study district was selected because it represented the geographical, and to some extent, the cultural diversity of the country. The district was also in close proximity to the Butajira district, which hosts study sites for Addis Ababa University.

### Selected priority disorders

Psychosis (including schizophrenia and bipolar mood disorder), depressive disorders (including depression with perinatal onset), alcohol use disorder, suicidality and epilepsy were the priority disorders. The disorders were selected because they carry a known high public health burden, and there is a reasonably strong evidence base for cost-effective interventions for their treatment. The prevalence of these priority disorders in Ethiopia is presented in Table 1.

**Table 1:** Prevalence of the selected priority disorders^a^

Disorder	Prevalence	Coverage
Schizophrenia,^12^ %	0.5	<1

Bipolar disorder,^13^ %	0.5	<1

Major depression,^14^ %	5.0	No data

Suicide		
Completed,^15^ per 100000/year	7.76	No data
Attempted,^16^ %	3.2	No data

Alcohol,^17^ %		
Problem drinking	2.2-3.7	No data
Dependence	1.5	No data

Epilepsy,^18,19^ %	0.5-2	2-13^b^

a. Figures are for lifetime prevalence except for depression where the figures are for 12-month prevalence and the other exception is suicide.

b. Only 4% of those not receiving treatment did not have the money to buy the medication. Default from treatment: 62% at 2 years.

### Development of the MHCP

**Fig. 1:**
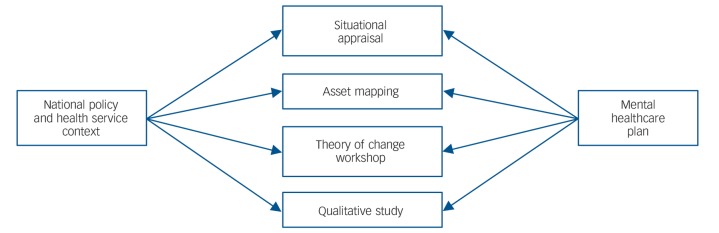
Formative work that informed the development of the mental healthcare plan.

The development of the MHCP was informed and guided by the HSDP-IV, the national mental health strategy and the mhGAP. Within these broader frameworks, a mixed methods approach consisting of situational appraisal, asset mapping, theory of change (ToC) workshops and qualitative studies (Fig. 1) were used to develop the MHCP.

#### Situational appraisal

A situational analysis tool^11^ was employed for collection of detailed cross-sectional data on health and factors that are likely to influence the health of the Sodo district population. The methods and results of this have been reported previously.^11^

#### Asset mapping

A community resource inventory^20^ was adapted to collect information on resources available in the community. The inventory assesses various domains of community resources, including physical assets (for example forests), community associations, health facilities, faith and traditional healers, education facilities, justice system, recreational venues, agriculture, religious institutions and non-governmental organisations (NGOs). Health extension workers were trained for 2 days before administering the inventory. On average each health extension worker interviewed two key informants to complete the inventory, including community leaders, subdistrict chairpersons, district officials, teachers and community elders. A district-level summary of community resources with a potential for use during the implementation of the MHCP were collated.

#### ToC workshops

Details of the methods are described elsewhere^21^ and in elsewhere in this supplement.^22^ Two pre-ToC meetings and two ToC meetings were undertaken. The first pre-ToC workshop was conducted within the PRIME team, where the main outcomes of the intervention, and the pathways required for these outcomes, were conditionally mapped. This was further developed in a country pre-ToC workshop in which the PRIME team members met on two separate afternoons to describe in more detail the necessary steps, the interventions and assumptions for reaching the outcomes. Then two ToC workshops were carried out with key stakeholders in the district and with national-level policy makers.

#### Qualitative study

In the qualitative study, we explored the feasibility and acceptability of integration of mental healthcare into primary care. We conducted both individual in-depth interviews and focus-group discussions involving key stakeholders targeted by the MHCP intervention levels: community, health facility and healthcare organisation. At the community level, we carried out one focus-group discussion each with health extension workers, health development army volunteers (network of volunteers that support community participation in health programmes) and families of people with mental and developmental disorders. We also conducted individual in-depth interviews with traditional and religious leaders (*n* = 5), community leaders (*n* = 3) and representatives from NGOs active in the area (*n* = 2). At the facility level, three focus-group discussions were conducted with primary healthcare front-line workers and supervisors. At the healthcare organisation level, we carried out four individual in-depth interviews with key stakeholders in the district health office and Federal Ministry of Health. Three research assistants (all Ethiopian women with Masters degrees in social work) conducted the interviews in Amharic, the official language of Ethiopia. We used standardised case vignettes (based on those used in the WHO key informant survey^23^ and used in Ethiopia previously^24^) to orientate respondents to the type of disorder under consideration. Individual in-depth interviews and focus-group discussions were audiotaped, transcribed in Amharic and then translated into English by the interviewers prior to coding. A framework analysis approach was utilised, following the recommended four-step procedures.^25^ The data were managed using qualitative data analysis software (ATLAS.ti, version 5.0 and Nvivo, version 9).

#### Modelling

Data from the various methods were triangulated to develop the MHCP. This is demonstrated in Table 2 and Table 3.

**Table 2:** Contribution of the various formative methods in the development of the mental healthcare plan (MHCP)

Method used in the developmen of the MHCP	Contribution of methods to the content of the MHCP
Situational analysis	Define context for intervention

Asset mapping	Define area of need (such as inclusion of alcohol use disorder) Identification of resources to support intervention

Theory of change workshop	Define desired outcome chains Define indicators to determine achievement of outcomes Define interventions to achieve goals and outcome Define how outcomes should be measured

Qualitative research	Define acceptability of MHCP Define capacity needs of staff Define the required resource management Define the support and supervisory needs of staff and patients

**Table 3:** Application of the triangulation of the various methods to inform the community package of the mental healthcare plan (MHCP)

Methods of MHCP input assessment	Findings from the method	MHCP response (package) resulting from the finding
Qualitative research	Need for emphasis on empowerment of patients and families of patients Need for stigma reduction activity Encouragement and spiritual support for patients and families Need to meet basic needs, not just offering medications	Individual empowerment through community-based rehabilitation Community awareness-raising package Engagement package with faith and traditional healers Community-based rehabilitation

Asset mapping	Community resources with potential for supporting care provision High level of potential risk factors (such as alcohol) Potential barriers, particularly geographic inaccessibility	Community partnership package (to engage and mobilise community resources) Inclusion of alcohol as target disorder Outreach support (home visit and support at the health post)

Theory of change workshop	Low level of mental health coverage with many people physically restrained (chained/tied up) at home Need for improving demand by community Gap in detection and referral Need for supporting engagement in care, for example concordance with medication	Community case detection package Community awareness-raising package Training package for community health workers and other providers Community engagement package (with focus on faith and traditional healers) and community-based rehabilitation

#### Piloting and ethical considerations

The facility training packages were piloted in one health facility – the Kella health centre – and acceptability of the packages was assessed. The health information system and cases seen over 2 weeks in two out-patient clinics were assessed. The key informant training for case detection of psychosis and epilepsy was also piloted with community leaders and health extension workers. The study was approved by the Institutional Review Board of the College of Health Sciences of Addis Ababa University. All participants in the different substudies provided informed consent.

## Results

### Sodo district health system context: results from situational appraisal and asset mapping

The district has 8 health centres and 58 health posts. The number of staff in the health centres, mostly nurses, ranged from 8 to 24 (Table 4). The average number of people served by each health centre in the district is around 20 000 and the average number of people served by each health post is around 3000. There are no hospitals or mental health services in the district. A large number of informal providers, mainly faith and traditional healers, including herbalists and spiritual healers (about 200) and a few NGOs were also identified as potentially important support providers.

**Table 4:** Sodo district health human resource profile of implementation health centres

	*n*													
Name of health centre	Nurse (diploma)		Nurse (BSc)		Public nurse		Midwife		Environmental health officer		Health officer		Pharmacy technician	
	Men	Women	Men	Women	Men	Women	Men	Women	Men	Women	Men	Women	Men	Women
Buei	5	4	2	1	1	1	–	2	–	1	3	1	2	1

Kella	6	5	2	–	1	2	–	1	–	1	2	–	–	1

Tiya	5	2	–	–	–	–	–	2	–	–	3	–	–	1

Gerino	3	1	–	–	–	–	–	3	–	1	1	–	1	–

Endebuye	5	1	–	1	1	–	–	1	–	–	–	–	1	–

Adele	6	–	–	–	1	2	–	2	–	1	1	–	1	–

Wella Wella Wedesha	6	2	–	1	1	–	–	1	–	–	1	–	1	–

Beke Bisan Jilba	3	2	–	–	–	–	–	1	–	–	1	–	–	1

### MHCP and intervention packages

The MHCP is presented for the three levels of the health system in the Sodo district: the community, health facility and the healthcare organisation levels. Further details of the PRIME intervention packages for the three levels of the district health system are provided below and in Table 5.

**Table 5:** Summary of key interventions at the various level of the system and expected short-term outcomes resulting from intervention

Level of intervention	Key tasks	Key intervention components	Expected short-term outcome from intervention
District administration (and higher administration)	Overall coordination and lead Mentoring and supervision Resource allocation	Sensitisation and advocacy Establish advisory board and 6-monthly advisory board meeting	Ownership of the integrated mental healthcare Support evaluation of the care Ensure sustainability

Healthcare facility (health centre)	Case detection/diagnosis Provision of treatment (medication and basic psychosocial care) Provision of gender-sensitive care (such as psychosocial risks; pregnancy, breastfeeding; alcohol)	Sensitisation of all staff Training of clinicians using mhGAP intervention guide and other manuals Decision support Job aid Well-being support	Non-stigmatising (inclusive) care Delivery of competent care Provision of continuing care Enhanced staff well-being

Community			
Health extension workers	Identify people with psychosis/ epilepsy and referral to health centre Perinatal support and referral Identify and refer alcohol use disorders Engage in care and adherence support Community training and advocacy Promotion of rights protection Encourage social inclusion	Manualised training Supervision/mentoring Well-being support	Non-stigmatising attitude Competent at detecting psychosis and epilepsy/alcohol/perinatal conditions and providing basic psychosocial support Ongoing community sensitisation health extension worker well-being
Health development army	Detection and referral of psychosis/ epilepsy to health extension worker Engagement in care, including adherence support Encourage social inclusion	Manualised training by health extension workers	Non-stigmatising attitude Competence at information dissemination
Faith and traditional healers	Detection and referral of psychosis/ epilepsy/alcohol to health extension worker or health centre Adherence support Encourage social inclusion Promote human rights protection	Manualised training by health development army/health extension worker	Non-stigmatising attitude and care Collaborative care, including referral
Community leaders	Detection and referral Encourage social inclusion Encourage treatment adherence Promote human rights protection	Manualised training by health development army/health extension worker	Non-stigmatising attitude and care Collaborative care, including referral
Community residents, non-governmental organisation	Non-stigmatising attitude Livelihood support	Training in community conversation, training by health development army brochures and posters	Non-stigmatising attitude Participation in livelihood support

mhGAP, World Health Organization Mental Health Gap Action Programme.

#### MHCP packages for the community level

The goal of the community-level MHCP intervention packages is to improve access and community inclusiveness. Four main intervention packages are included in the MHCP: community awareness raising and stigma reduction; community case detection; support to continuing care; and community-based rehabilitation.

**Community awareness raising and stigma reduction**. Awareness raising is aimed at improving knowledge regarding the nature of mental disorders, their aetiology, case detection, service utilisation, stigma reduction and discrimination, protection of human rights and dignity, and social reintegration. The interventions are delivered through simple information leaflets and posters that are distributed through the district, through workshops, community conversation meetings and social associations. Awareness-raising events are held by health development army members and health extension workers.

**Community case detection**. Health extension workers are ideally placed to improve case detection in the community because of their obligation to visit each household in their catchment area (500–1000 families) every 3 months; their links with community leaders; and their close relationship to the health facility (including monthly meetings at the health centre, supervised by health centre staff). This approach to case detection fits closely with their expected roles and responsibilities for other disorders, and with the national mental health strategy. The disorders amenable for this type of case detection are psychosis and epilepsy. Given previous experience in case detection in other studies in Ethiopia,^26^ health extension workers will be supported by community leaders.

**Continuing care and community-based rehabilitation**. Health extension workers will be trained to provide continuing care support primarily for people with psychosis and epilepsy and their families. Tasks to be carried out by health extension works during their visits every 3 months include the following.

- Psychoeducation about the illness:
  - raising awareness of the need for continuing care even when the person is symptom free;
  - safe management of aggressive behaviour;
  - mental health first aid/first aid for seizures.
- Monitoring medication side-effects, providing adherence support and referring for review if needed.
- Monitoring of mental state, detecting early signs of relapse and referring for review when needed.
- Asking about physical health and supporting access to health facility for assessment and care.

This additional support will only be part of their routine home visitations.

For severe mental illness in mothers, in addition to the above, the care package considers the physical health needs of the mother (such as accessing routine antenatal and postnatal care and physical healthcare for illness), social support needs of the mother (mobilising additional support) and welfare of the children (in terms of risk, but also accessing routine healthcare for vaccinations and prompt treatment of illness). For people with schizophrenia who fail to respond to 6 months of standard MHCP, community-based rehabilitation is provided. The main functions of community-based rehabilitation are as follows.

- Rehabilitation, for example supporting self-care.
- Community mobilisation to support:
  - social inclusion and involvement in community activities; and
  - support for families (financial, food, practical support).
- Multisectoral collaboration to access opportunities for skill development and livelihoods support.
- Adherence support.
- Family intervention (emotional support and encouraging supportive interactions/discouraging high expressed emotion within the home).
- Detection of side-effects, inadequately controlled symptoms or relapse.

#### MHCP packages for the healthcare facility level

The focus of the PRIME intervention is to build capacity for competent and inclusive care in the healthcare facilities (health centres). PRIME MHCP has four intervention packages to achieve this: training of clinical staff; sensitisation workshops (interactive workshops, including testimonial from patients, involving all staff of the health facilities); supervisory decision support; and support of staff well-being.

**Training of clinical staff**. This is aimed at enabling case detection (assessment and diagnosis, including identification of psychosocial risks such as domestic violence), prescription of psychotropic medications, provision of basic psychosocial care, referral and ongoing care. Training was provided for 2 weeks: 1 week theoretical training and 1 week clinical apprenticeship at a psychiatric clinic (3 days) and at a health centre (2 days). The core training tool is the mhGAP intervention guide.^27^ The delivery of the mhGAP intervention guide training was modified with additional training components, namely: (a) the training was provided for longer periods of time; (b) practical training included encounters with actual patients with mental health needs; (c) on the job support and supervision was provided for 1 additional month to ensure competent care; and (d) explicit training was added on maternal depression and the continuing (chronic) care model. Moreover, culturally adapted psychosocial intervention packages and intervention packages for alcohol use disorders were not available and a decision was made to develop these packages for a phased delivery of care in the future.

**Sensitisation of all staff**. All staff, including non-clinical staff, were given half-day interactive workshops to enhance awareness and inclusiveness. Training used case vignettes and testimonials from patients.

**Decision support**. Finally, the MHCP mandated provision of additional decision support mechanisms: pocket guides, posters and supportive supervision. Two forms of supportive supervision are incorporated. The first is an integrated supervision, which involves using staff who are designated to be supervisors within the health system. The second approach uses specialist (psychiatric nurse) supervisors.

**Health professional well-being**. The final intervention package focused on improving health professional well-being. There are no culturally adapted intervention packages for supporting staff well-being and this will be developed following a comprehensive evaluation of the well-being of health professionals.

#### MHCP packages for the healthcare organisation level

The key intervention packages were programme management through proactive stakeholder engagement, advocacy and sensitisation. The stakeholders engaged in the healthcare organisation included those at the Federal Ministry of Health, and at the regional, zonal and district health administration bureaus and offices. Two structures were developed to support stakeholder engagement: a country management group and community advisory board. The country management group had seven members representing the Ministry of Health, the regional health bureau, zonal health bureau, district health office, a mental health coordinator of the district and two of the PRIME country investigators. The community advisory board had 17 members and was represented by key members of the district leadership (security, gender office, women and youth affairs, religious affairs and education), the community and a carer, and was chaired by the head of the district health office. Meetings every 6 months were held with both the country management group and the community advisory board. Each meeting lasted half a day and activities of the preceding 6 months and plans for the future were discussed in detail. These meetings were also for advocacy and sensitisation of the district leadership. To further support programme management, the district has allocated a mental health coordinator, funded by the Ministry of Health. This will be followed by allocation of mental health coordinators at the healthcare facilities. Other programme support mechanisms have included the training of trainers, which is being coordinated by the district. Two health officers are trained to be trainers.

### Acceptability of integrating mental healthcare into primary care and making it work

Here we consider the summary results of the qualitative study. In broad terms, participants considered the integration of mental health into primary care to be feasible and acceptable. However, three main preconditions were considered essential for the provision of integrated care. First, access to adequate staffing and training; second, providing ongoing supportive supervision to clinical staff; and third, focus on recovery and the basic needs of patients in relation to food, shelter and clothing.

The emphasis in relation to developing the skills of staff to provide competent care was on averting potential negative consequences of poor skills. Perceived inability of staff to provide competent care could discourage them from taking on tasks that are considered a specialist area. Mismanagement of patients could put the patient and the community at risk of harm, which may eventually lead to mistrust of the service being provided and reduce motivation to access care. Related to this, the need to increase the number of staff was emphasised given the potential to increase the service load substantially in the longer term.

The second theme of supportive supervision was also considered essential to evaluate the implementation process, to address emerging challenges and to support skill development in the area of patient assessment, care provision and clinical records. Ongoing and integrated supervision was emphasised. The participants proposed that the supervision should be offered as long as integrated mental healthcare is being provided.

The third theme, ensuring recovery, was considered the core of care provision. Essential elements for recovery were: addressing basic needs, ensuring treatment adherence, supporting family, engagement with the community and reducing social exclusion. There was consensus that medication was not adequate to bring the person with mental illness back to their previous state of health because, in the words of one participant, they ‘have lost everything because of chronic illness’. Interventions should address the basic needs for food, shelter and livelihoods. Economic security, protection from stress, moral support and encouragement were considered essential components. The potential for learning from other programmes such as HIV/AIDS were discussed but participants were cautious, saying that large investment accompanied the HIV/AIDS programmes and that is difficult to replicate in other programmes. Ensuring medication supply and treatment adherence were the other issues. Participants proposed that the network of health extension workers and the health development army would be able to support treatment adherence and to invite patients back to care. The role of families should be recognised and their needs for both practical and emotional support met. Families carry a huge emotional burden caring for their loved ones. Families should be equipped to support and to provide a therapeutic environment for the individual with illness. The community was considered an essential part of the support mechanism for the patient and family. The participants acknowledged the long history of people supporting each other in rural Ethiopia. Nevertheless reliance on an ‘overstretched community’ to mobilise the necessary resources may not be feasible. Carers particularly underscored the limited capacity of the community to keep on giving after the initial crisis has passed and their disinclination to feel dependent on others who already had their own difficulties. Encouraging social inclusion within the community was important to achieve recovery. Mechanisms mentioned by participants that could encourage social inclusion were: public contact with people with severe mental illness, including discussions of mental illness in social gatherings, educating the community about the treatability of mental disorders and the involvement of people with mental illness in social activities and decision-making.

### Delivery of the MHCP

**Fig. 2:**
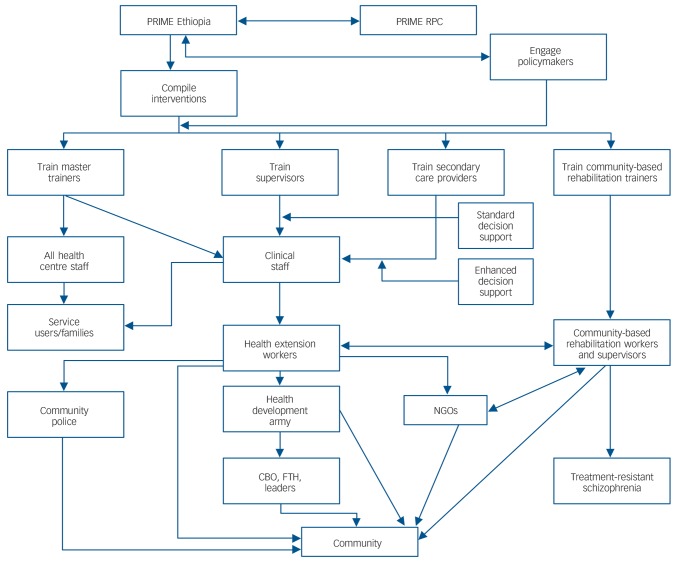
Cascading delivery of the mental healthcare plan. Standard decision support involves integrated supervision and general decision support tools. Enhanced decision support involves supervision by psychiatric nurses in addition to the general decision support tools.PRIME, PRogramme for Improving Mental health carE; PRIME RPC, PRIME Research Programme Consortium.

The MHCP also included plans for how the intervention packages should be systematically cascaded from the PRIME team, with the support of the district administration and policy makers down through various providers to users and participants in the community (Fig. 2).

### Pilot results

Pilot results describe the acceptability of the training, cases seen and key informant identifications. At the pilot site, 19 primary care staff (nurses and health officers) were trained. All the topics covered had very good acceptability. The methods of training that the trainees found particularly useful were the participatory methods, the case scenarios, the video clips and the practical training. In the practical training at the psychiatric clinic in Butajira, trainees saw over 200 patients. In addition to cementing their knowledge, the practical training had an impact on their attitudes. First, trainees appreciated the public health dimension of mental disorders, for example that mental disorders may be more common than they thought. Second, the trainees were encouraged to see the improvements of patients with treatment. As one participant said, ‘If we provide mental healthcare, we can change the life of people in need’. Some of the trainees expressed their interest in learning and practising psychiatry full time. For example, one health officer said to the psychiatric nurse supervisor, ‘I would love to get this opportunity (to specialise in psychiatry)’.

In the 2 weeks after the training, two health officers working in the out-patient clinics had seen 24 patients. One of the health officers said, ‘It is enjoyable when you do the work’, particularly when ‘people have confidence to talk to you’ about their difficulties. ‘People who come to see me are lucky’ because she now has a more ‘complete knowledge’. She found that using the mhGAP intervention guide in the clinic gave her confidence to make a diagnosis and provide treatment. Although some individuals struggled to accept the diagnosis of a mental illness and opt to go for traditional treatments, most attended for follow-up. For example, nine of the ten individuals with appointments attended for their appointment. Having the service nearby has also encouraged attendance for follow-up. What the staff find crucial is the time taken to explain the illness and treatment. One of the health officers said she often makes appointments to speak to the patients at the end of the clinic. The conditions seen by two health officers is presented in Table 6. Although the selected priority disorders, except epilepsy, are not shown in the health management information system (HMIS), the zone and the district permitted recording of the selected conditions as non-HMIS disorders. The district administration also expressed its satisfaction with the capacity development work. However, to facilitate supply of medications, the district requested PRIME to provide an estimate of how much medication would be required. Therefore, the PRIME Ethiopia team purchased a 3-month supply of drugs for the pilot health centre to get data on required amounts of medications.

**Table 6:** Conditions seen over 2 weeks at the pilot site

	*n*	
Diagnosis	Men	Women
Psychoses	1	5

Depression	1	5

Suicidality	0	3

Mild depression and other disorders	4	4

Alcohol use disorder	2	1

Total	8	18

Two groups of key informants were trained: community leaders (*n* = 13) and health extension workers (*n* = 15). The half-day training focused on the identification of severe mental illness and epilepsy. After the training, key informants were asked to give a list of potential patients with the two conditions. Health extension workers identified more cases than the community leaders for both conditions. For severe mental illness, health extension workers identified 67 potential cases with a male to female ratio of 2.7:1 and community leaders identified 33 cases, with a male to female ratio of 4.5:1. With regard to epilepsy, health extension workers identified 25 cases (male to female ratio of 2.6:1) and community leaders identified 15 cases (male to female ratio of 14:1).

## Discussion

The MHCP presented here described the intervention packages considered essential for the provision of a functioning integrated mental healthcare system in rural Ethiopia. These packages are anchored in three principles: the need to ensure accessibility; enhancing capacity of facilities and leadership; and the need to ensure sustainability.

### Ensuring accessibility

Most people in the Sodo district live in scattered rural villages that are difficult to access. The people hold traditional views about the causation and treatment of mental disorders. Thus, ensuring geographical and cultural accessibility of the services was essential. In line with this consideration, the MHCP proposed systematic engagement with the community, community opinion leaders and policy makers and traditional healers. Community sensitisation packages, engagement with traditional healers and community organisations are likely to improve acceptability of services and social inclusion. The MHCP recognises the role of families and the need to support them, as was demonstrated in the qualitative study.

### Facility and organisational capacity

Staff at the facility should provide safe and competent care that enhances inclusiveness and acceptability. The sensitisation workshops that involved all the staff were provided to ensure inclusive care in the facilities. The mhGAP intervention guide has been, encouragingly, accepted by trainees and appears to encourage confidence. However, the additional practical training, which is not part of the mhGAP intervention guide, were considered very important by trainees in helping them understand the public burden of mental disorders and its treatability, and also in motivating them to provide care. This model may have utility for other similar settings. Preliminary findings also suggested that the service provided may be feasible and acceptable. Although pragmatic, given existing resource constraints, it is unclear whether supervision by non-specialists can work in practice. Hence the Ethiopia PRIME study will include a future evaluation of the impact of the existing supervisory structure utilising non-specialists compared with enhanced supervision delivered by psychiatric nurses. However, more adaptations are likely to be required as the implementation of the MHCP progresses.

### Sustainability

All aspects of the MHCP were designed with sustainability in mind. The involvement of key decision makers and the community in developing the MHCP, the establishment of coordinators, training structures (Fig. 2) and the engagement of community resources and families in the plan are all meant to encourage sustainability. Engaging stakeholders is particularly important when programmes being proposed have the potential to introduce new and large demands on the system in a sustained way. The integration plan has the potential to permanently change the resource requirements and the workload of the staff and the district. The programme will require substantial cultural change among the providers and management. One of the strengths of the PRIME project in Ethiopia has been the full participation of the district decision makers. The policy commitment of the government, as indicated by the mental health strategy and the various initiatives of the Federal Ministry of Health, makes this the most opportune time to implement integration and scale up. Yet one of the main threats for the project will be the failure of the project to sustain this commitment from the district or failure to replicate the level of engagement in future scale up. A related threat is changes in leadership and turnover in staff. This requires vigilance, continuous monitoring and a rapid and robust response. The ultimate success depends on the district taking full ownership of the mental health programme as an integrated care provision. It also will depend on affordability and on factors that go beyond the district and are influenced by broader policy issues. For example, the integrated programme is more likely to be successful if this becomes the norm across the country. Current policy directions indicate that integrated care will be the norm throughout Ethiopia. Having trainers from the district itself will ensure training is provided on a continuous basis given the relatively high turnover of staff.

### Implications of the pilot results

The pilot findings are encouraging. First, the support of the district for the pilot work is indicative of the commitment of the district for the provision of integrated care moving forward. Second, the acceptability of the training packages, particularly the practical training, is an important indication of potential success. Third, the patients' acceptability of the care is encouraging, given that most had returned for follow-up. Fourth, trained staff found delivery of the care gratifying. Finally, the key informant method has the potential to improve case detection and access. Health extension workers appear the most promising informants and are able to detect more women with potential disorders than the community leaders did. However, in both cases women are under-represented. This contrasts with cases detected at the health facilities where more women seem to be detected and treated (Table 6). Further work is needed to validate the key informant methods.

### Innovations

Perhaps the main innovation of the PRIME work in Ethiopia has been the deliberate and systematic approach PRIME took to understand the contextual factors relevant for provision of integrated care and the willingness to learn from local stakeholders. In this regard, for the PRIME Ethiopia team, the most important contributor to the MHCP development and implementation was the community advisory board. The community advisory board allowed broad (and grass roots) participation of the district leadership and political buy-in. The second innovation was the willingness to look beyond the biomedical domain, into what is available in the community. This helped us to map potential community resources that may support the provision of sustainable care to people with mental illness and their families. Related to this is the focus on the community for continuing care and community-based rehabilitation. The fourth innovation is the modification of the mhGAP training to include practical training, which was found to be a useful addition in consolidating knowledge and skill, and in having an impact on attitude. Finally, additional interventions in future will focus on improving the well-being of health professionals. Staff turnover is high in health centres in Ethiopia. Although not formally studied in the Ethiopian setting, many risk factors for staff burnout are present, including high patient loads, poor quality of facilities and lack of available interventions (engendering a sense of helplessness).

### Limitations

Although the MHCP is comprehensive, several components are yet to be piloted. Further evaluation is required to identify exactly what components of the intervention packages are feasible, acceptable and working. For example, providers in the folk sector are fully mapped, but how the relationship between the biomedical providers and the folk providers functions, and the impact of the evolving relationship on the delivery of care, has to be understood. The pilot data on training is limited to staff trained in one health centre. The actual patient data are limited to two health officers, who were trained at a pre-pilot stage. The key informant method of case detection requires further validation. It appears that, as it stands currently, this method has the potential to lead to inequitable care provision.

### Implications for the future

This study has the potential to make a substantial contribution to the scale up of mental healthcare in Ethiopia. But the success of the project will depend on whether the district, and ultimately the government, owns the integrated care approach. This will also depend on whether integrated mental healthcare is affordable and not just effective. Therefore, while introducing the MHCP, careful costing and exploration of other delivery options need to be looked into. For example, the integrated supervision may be a cheaper alternative if it is nearly as effective as the more expensive specialist supervision provided by psychiatric nurses. A larger-scale study, for example, nested during the scale-up phase of PRIME, may need to be conducted. Although community mental health service provision^28^ as it is known in the West is not feasible, PRIME is working to support integration of mental healthcare into the work of community health workers. The role of psychiatrists will also need to be redefined^29^ so that they focus on service development and supporting policy.
